# Hereditary Sensory and Autonomic Neuropathy Type IV in 9 Year Old Boy: A Case Report

**Published:** 2016

**Authors:** Mohaddeseh AZADVARI, Seyedeh Zahra EMAMI RAZAVI, Shahrbanoo KAZEMI

**Affiliations:** 1 Specialist of Physical Medicine and Rehabilitation, Department of Physical Medicine and Rehabilitation, Tehran University of Medical Sciences, Tehran, Iran

**Keywords:** Hereditary sensory autonomic neuropathy, Recurrent ulcer, Pain insensitivity, Congenital

## Abstract

**Objective**

The Hereditary Sensory and Autonomic neuropathy (HSAN) is a rare group of neuropathies that affects the Sensory and Autonomic nervous system. The patients do not have the ability of sensing different sensations such as pain and temperature, which tends to lead to different injuries. In addition, due to autonomic involvement, the patients suffer from fluctuation in body temperature periodically and lack of precipitation. HSAN is divided into 5 types according to the age of onset, clinical features, and inheritance. Our case was a 9-yr old boy from cousin parents. He had some developmental delay and history of recurrent fever and convulsion in the first year of his life. Gradually, other symptoms added to patient’ feature such as multiple painless skin ulcers, tooth loss, destruction of toes and fingers. In electrodiagnostic study, we found decreased amplitude of sensory nerves, while the other studies were normal. Laboratory test and imaging studies were also normal. All clinical and paraclinical findings were in favor of HSAN type IV. There is no cure for such patients; as a result, these patients and their families need receiving appropriate education and timely rehabilitation services.

## Introduction

Hereditary Sensory Autonomic Neuropathies (HSAN) are a group of rare hereditary neuropathies first described by Dearborn in 1932 as the “Congenital pure analgesia” (1). This neuropathy affects the sensory and automatic nervous system. HSANs are categorized into six main types based on the onset age, clinical features, and inheritance. Besides, electrodiagnosis (2) and 12 HSAN disease–causing genes have been identified (3).

In type 1, the onset age is between 20 and 40 yr and the inheritance type is autosomal dominant. The patients’ symptoms demonstrate as the reduction or lack of sensing pain and temperature in the lower limbs, which leads to sole ulcers and burning foot pain. The autonomic changes are not salient in this type. Frequent and repeated osteomyelitis may result in Charcot joints. In the electrodiagnostic studies of these patients, all sensory and motor findings are normal or have a slight reduction of amplitude and sympathetic skin response and quantitative sensory testing are abnormal. Except type 1 the inheritance of other type is autosomal recessive.

In type 2, the age of onset is from early infancy to childhood. The symptoms involve severe losing sensitivity of all patients’ senses such as touch, compression, and vibration. The painless destruction of distal phalanges, and undiagnosed fractures, are among other symptoms of the disease. In addition, sweating disorders and bladder complications happen in these patients. In electrodiagnosis study, the sensory responses are absent in most of the cases, sometimes, however, it has reduction of amplitude. Compound muscle action potential (CMAPs) is usually normal or slight reduction of amplitude. Quantitative sensory testing (QST) for vibration sense is disrupted.

In type 3, alternatively described as familial autonomy and seen rarely, the age of onset is infancy. The autonomic symptoms include temperature and blood pressure fluctuations, absence of tear, repeated lung infections, gastrointestinal disorders, hyperhidrosis, taste problems, and loss of corneal reflex. In the electrodiagnostic study of the nerves, there are slight reduction of sensory nerve action potentials (SNAPs) amplitude and slight prolongation in motor nerve conduction velocity. In addition, QST is abnormal and the sympathetic skin response (SSR) is normal.

In type 4, the disease symptoms start from infancy and are accompanied by pain sensitivity disorders, mental problems, and anhydrosis. In the electrodiagnosis study, there is slight reduction of the amplitude of SNAP and CMAP, and the QST is abnormal while SSR is normal.

In type 5, with insensitivity to pain, the patient experiences pain sensitivity disorder, while the rest of the autonomic problems do not exist. Due to the conflict of the fibers carrying the pain sense, in the electrodiagnostic study, all the tests are normal. In type 6, the patient present with dysautonomia; hypotonia; facial deformity; decreased pain response; joint contractures; retardation; respiratory failure; early death (4).

## Case Report

**Fig 1:**
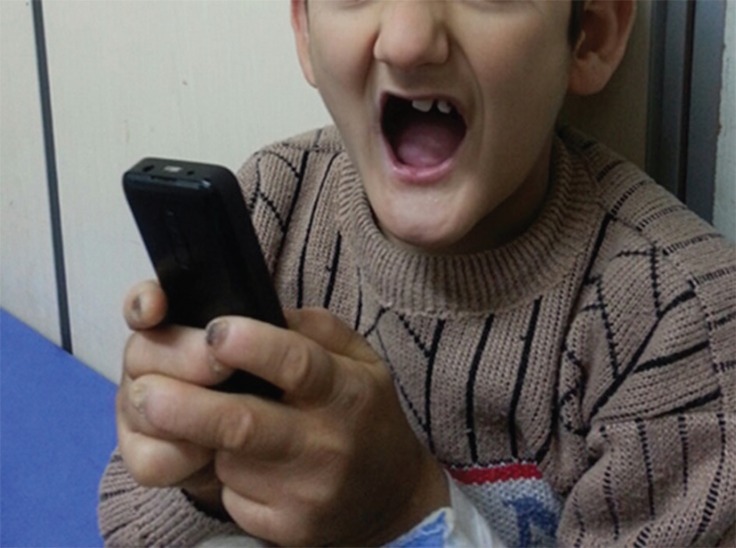
Loss of teeth

Our patient was a 9-yr old boy from a Kurdish family who was the ninth child of the family born in a natural delivery from cousin parents. The first six children of the family were healthy, while the seventh and eighth children died 2 to 3 months after birth for a reason for which the mother was unaware. The patient experienced developmental problems from birth. Gradually, he developed numerous skin ulcers and loss of teeth (Fig. 1). The patient experienced convulsion subsequent to fever at the age one yr. This has been repeated a number of times.

**Fig 2:**
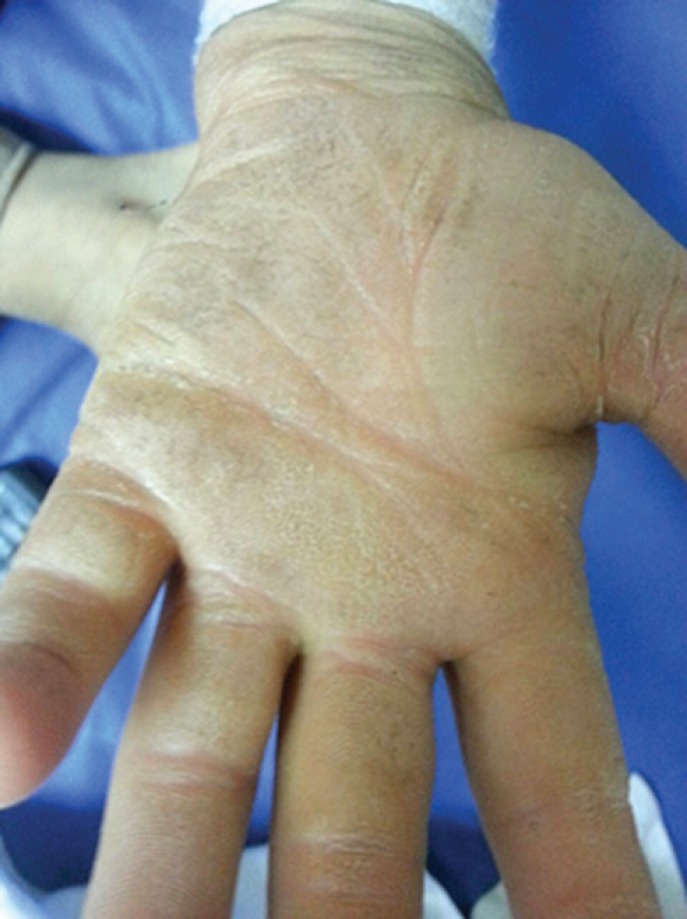
Hand’s hyperkeratosis

**Fig 3:**
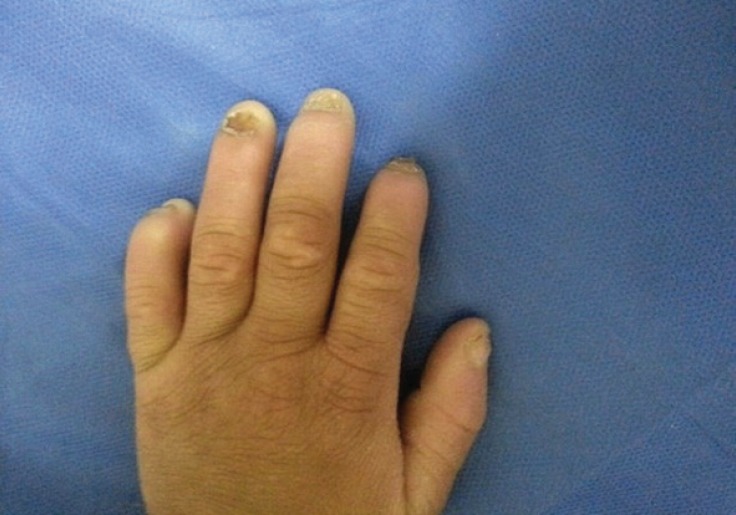
Fingers destroyed automatically

With the start of phenobarbital, the patient’s convulsion was controlled. However, his fever was occurred frequently. The patient had lacked perspiration from birth, and had had very little responsiveness towards painful stimulation. The palm and sole skin has gradually thickened and his fingernails and toenails have been deformed, and the fingers destroyed automatically (Fig. 2 & 3). His intelligence is slightly below normal, and he was studying in the 2nd grade of primary school. The patient did not have a proper eye-contact communication, and had poor cooperation in the examination. His gait was normal and so was the strength of the muscles. 

The deep tendon reflexes had decreased, and he had reduction of the sensitivity of heat and pain. He had no proper cooperation with regard to the vibration. His crying was tearless. 

In the conducted studies and examinations on the patient, all blood tests, including uric acid, was normal. The brain magnetic resonance imaging (MRI) was normal as well. 

In nerve conduction study, all CMAPs values were normal, except for a slightly reduced motor nerve conduction velocity in median and ulnar nerve at 46.5 m/s and in proneal and tibial nerve at 44 m/s (normal range in median and ulnar nerve >50.5 m/s and in tibial and proneal nerve >48 m/s).

The SNAPs of median, ulnar and sural nerve were significantly decreased, in right median nerve (3μV, normal range >7 μV) and in left sural nerve (5μV, normal range> 13 μV).

In the electromyography, no findings were discovered except for reduction of recruitment in the distal muscles of upper and lower limbs. In genetic study found mutation in NTRK1 gene. Considering the clinical symptoms, age of onset, and electrodiagnostic test, in addition to the autosomal recessive inheritance (familial marriage of parents) and genetic study, the type of the patient’s disease was HSAN Type IV.

## Discussion

HSAN IV disease is caused by mutation of NTRK1 gene on the 3q chromosome (5). This disease usually occurs with lack of sensitivity in all types of senses, but our patient had reduction of sensitivity towards heat, and pain; therefore, he had not lost all of these senses and sensitivity. He could distinguish hot and cold stimulants with delay. In HSAN IV, degenerative arthritis, recurrent osteomyelitis, automatic amputation of fingers and toes, and Charcot joints occur. Our patient was afflicted with recurrent ulcers leading to chronic osteomyelitis, and automatic amputation of the fingers. However, fortunately, he had not developed Charcot joints yet. In the electrodiagnostic study, the results were in the form of lack or reduction of sensory responses amplitudes in the presence of normal motor responses, and reduction or loss of autonomic response in the SSR test. The results of our patient were based on this finding. However, regarding the clinical evidence and hereditary issues, the disease can show heterogeneity and has a number of symptoms from different types of the disease (6). Our patient had a number of symptoms from other types of HSAN, for example, he had tearless crying which is most prevalent in HSAN Type 3 (7). 

The patient suffers from teeth loss, caused by numerous damages by the patient’s manipulation himself. 

Dental and oral complications of these patients start with growing the teeth in the form of biting the lips, manipulating, and extracting the teeth. Rehabilitation in fields of dental and oral issues can be very significant to these patients (8). In the past, the remaining teeth were also extracted to prevent further damage, however, nowadays use of dental prostheses and training patients are highlighted and accentuated in the treatment plan (9). 

Our patient is suffering from infectious ulcers, which are resistant to treatment in the feet. As previously mentioned in other articles, the disease should be considered in the differential diagnoses of patients with recurrent limb ulcers, with healthy immune system (10).

Self-mutating behavior happens in these patients, caused by the reduction of pain sensitivity. Sometimes, however, it may cause a pleasant sense instead of sense of pain (11). In our patients, there were such behaviors, which had led to extraction of teeth and mutation of the distal phalanges of fingers.

**In conclusion, **since the disease is congenital, there is no specific and deterministic cure for it. There is need, for the supportive and preventive measures to ease and reduce complications. Patients and families training to prevent ulcer creation, and timely treatment are necessary. In addition, patients’ rehabilitation, prescribing the necessary supportive tools including orthotics and prosthesis are among the main priorities of treating such patients. 

## Notes

**Ethical Approval: **Not applicable.
